# Targeting the Small GTPase Superfamily through Their Regulatory Proteins

**DOI:** 10.1002/anie.201900585

**Published:** 2020-01-30

**Authors:** Janine L. Gray, Frank von Delft, Paul E. Brennan

**Affiliations:** ^1^ Structural Genomics Consortium University of Oxford, NDMRB Old Road Campus Oxford OX3 7DQ UK; ^2^ Target Discovery Institute Nuffield Department of Medicine University of Oxford Old Road Campus Oxford OX3 7FZ UK; ^3^ Diamond Light Source Harwell Science and Innovation Campus Didcot OX11 0QX UK; ^4^ Alzheimer's Research (UK) Oxford Drug Discovery Institute Nuffield Department of Medicine University of Oxford Oxford OX3 7FZ UK; ^5^ Department of Biochemistry University of Johannesburg Auckland Park 2006 South Africa

**Keywords:** drug discovery, peptides, protein–protein interactions, small GTPases, small molecules

## Abstract

The Ras superfamily of small GTPases are guanine‐nucleotide‐dependent switches essential for numerous cellular processes. Mutations or dysregulation of these proteins are associated with many diseases, but unsuccessful attempts to target the small GTPases directly have resulted in them being classed as “undruggable”. The GTP‐dependent signaling of these proteins is controlled by their regulators; guanine nucleotide exchange factors (GEFs), GTPase activating proteins (GAPs), and in the Rho and Rab subfamilies, guanine nucleotide dissociation inhibitors (GDIs). This review covers the recent small molecule and biologics strategies to target the small GTPases through their regulators. It seeks to critically re‐evaluate recent chemical biology practice, such as the presence of PAINs motifs and the cell‐based readout using compounds that are weakly potent or of unknown specificity. It highlights the vast scope of potential approaches for targeting the small GTPases in the future through their regulatory proteins.

## Graphical abstract

**What a PAIN**: Small GTPases have been notoriously difficult targets for small molecule and biologic therapeutics. This review explores the molecules targeting the GEF, GAP, and GDI GTPase regulatory proteins. It identifies issues in the chemical strategies, including PAINs motifs, insufficient potency, and lack of selectivity, whilst also providing thoughts on how this field will develop in the future.

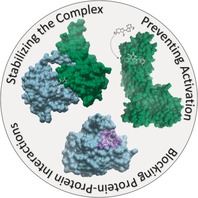

## 1 Introduction

**Figure 1:**
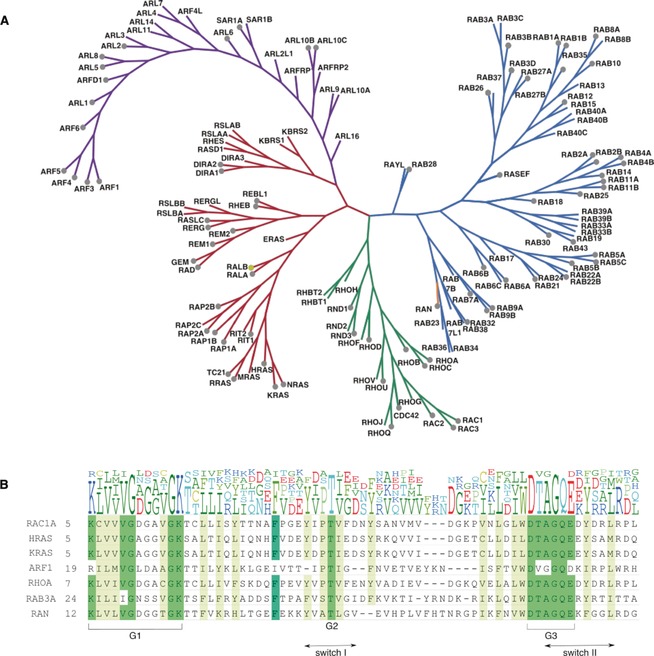
A. Phylogenetic tree of the Ras superfamily. Nine unclassified members are not included. The Rab subfamily is shown in blue, Ran in orange, Rho in green, Ras in red and Arf in purple. Grey dots indicate the X‐ray crystal structure has been solved. A yellow dot indicates an NMR structure. B. Comparison of different subfamily sequences containing the switch I and switch II regions. Characteristic “G‐boxes” G1, G2 and G3, which are involved with nucleotide and magnesium ion binding, are mainly conserved across the subfamilies. Whilst some key residues are maintained across the superfamily, the divergence in sequence between the subfamilies could be exploited for selectivity by therapeutics.^1^

The Ras superfamily of small GTPases are guanine‐nucleotide dependent molecular switches involved in the regulation of numerous cellular processes.^1^ With over 150 family members, the superfamily can be split into five smaller, evolutionally conserved subfamilies—Ras, Rho, Ran, Rab, and Arf—based on their sequence, structural similarity, and functions in the cell (Figure 1).^2^ The Ras subfamily, comprised of 36 members, is responsible for the regulation of signaling pathways involved in cell proliferation, morphology, and differentiation, as well as cell survival. The 20 members of the Rho subfamily are key regulators of actin organization, gene expression, and cell cycle progression. The largest subfamily, Rab, consists of over 60 members and is involved in vesicle and protein transport. The Arf proteins, with 30 members, are also regulators of intracellular trafficking. The Ran subfamily consists of only one protein, which is the most abundant small GTPase in the cell and is involved in nuclear transport.

Ras proteins cycle in the cell between an inactive GDP‐bound form and an active GTP‐bound state, whereupon the GTPases can bind to effectors and regulate cellular processes. GTPases modulate their effector proteins through a variety of methods, including inducing conformational change for substrate binding, relief of autoinhibitory intramolecular interactions, and translocation to membranes.^3^ Upon exchange of GDP to GTP, there are conformational changes in the switch I and II regions of the GTPase, which results in the GTPase having high affinity for effectors.^4^

**Figure 2:**
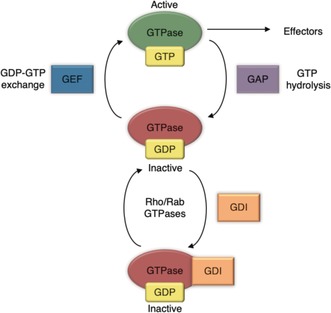
Small GTPase activation cycle with GEFs, GAPs, and GDIs.

This cycle is tightly regulated by guanine nucleotide exchange factors (GEFs), GTPase activating proteins (GAPs), and, for the Rho and Rab sub‐families, guanine nucleotide dissociation inhibitors (GDIs) (Figure 2). GTPases bind to the guanine nucleotides GDP and GTP with picomolar affinity and a very slow off‐rate, so the intrinsic rate of nucleotide exchange from GDP to GTP is inherently too slow for rapid control of signaling processes in the cell. GEFs catalyze the dissociation of GDP, allowing GTP to bind instead, and thus produce the active form of the GTPase. Conversely, GAPs stimulate the hydrolysis of GTP to GDP, leading to the inactive form of the GTPase and preventing it from being constitutively active. GDIs bind to and sequester the inactive form of the GTPase away from cellular membranes. This prevents the dissociation of GDP or the interaction with effector molecules, which provides an additional level of control over the activity of the Rho and Rab subfamilies.^5^

Owing to their vital roles in the cell, dysregulation of the activity of the small GTPases has been linked to a wide range of diseases. The most famous examples are the Ras proto‐oncogenes HRas, KRas and NRas, which are mutated in circa 25 % of cancers.^6^ Whilst these small GTPases have been extensively studied, other superfamily members are also linked to disease, including cancer, neurodegenerative, and autoimmune diseases.^7^ Disorders can arise from abnormal regulatory activity; for example, overexpression or mutations of GEFs, GAPs, or GDIs are linked to several forms of cancer and neurodegenerative diseases.^8^

Members of the family have been identified as important biomarkers in different disease types. Experimental research using mutant studies of GTPases have been hampered by the lack of chemical probes, and complex signaling pathways, with some negative mutants of the GTPase being able to bind to effectors and regulators, leading to misleading interpretations.^9^

Owing to their ubiquitous presence and roles in almost all cellular processes, the Ras superfamily and their regulators have become of paramount importance in the development of therapeutics and chemical probes.

However, the Ras proteins have come to be considered as “undruggable”. Medicinal chemists were originally inspired by the successful development of nucleotide analogue inhibitors of the ATP‐binding site of kinases to attempt a similar strategy for the GDP/GTP‐binding site of GTPases. Kinases bind to ATP with micromolar affinity (with ATP concentrations in the cell being in the low millimolar range) whilst the GTPases have picomolar binding affinity for their guanine nucleotides. This, in combination with the high micromolar concentrations of GDP (>30 μm) and GTP (>300 μm) in the cell,^10^ has resulted in unsuccessful attempts to develop sufficiently potent or selective nucleotide competitive inhibitors, apart from recent covalent inhibitors specifically targeting KRas G12C.^11^ Aside from the nucleotide‐binding pocket, the surface of Ras proteins is relatively smooth, with exploitable allosteric pockets rarely identified. This is perhaps not unexpected, as Ras proteins are mainly involved in protein–protein interactions (PPIs). PPIs have historically been difficult to target with small molecules owing to intractable large shallow surfaces. Alternative methods of inhibition, such as farnesyltransferase inhibitors (FTIs) and geranylgeranyltransferase inhibitors (GGTIs), which attempted to interfere with membrane localization of the GTPases, were pursued but failed to progress in clinical trials owing to inaccurate preclinical models, off‐target inhibition, and toxicity.11a, 12 A more recent strategy involved targeting downstream effectors of the GTPases in the signaling pathways. This has been somewhat successful, with inhibitors of kinases downstream of Ras being approved (such as vemurafenib).^13^ However, this method has also been associated with difficulties such as selectivity, complex feedback mechanisms, and the development of drug resistance.11a, 14

Novel methods of targeting the Ras superfamily are required to develop chemical probes and therapeutics. Recent reviews have detailed a general overview of methods to chemically target the superfamily (or for a single subfamily), including targeting GTPase‐effector interactions, covalent modifications, and directly targeting the guanine nucleotide binding site.14a, 15 Other reviews have concentrated on therapeutic strategies for a subfamily in association with a particular disease type. This review will concentrate in detail on the small molecule and biologic attempts to inhibit the Ras superfamily through targeting their modulators, the GEFs, GAPs and GDIs. This review will also highlight issues identified in the chemical strategies, including the reports of small molecule binders that contain pan‐assay interference (PAINs) motifs or toxicophores/structural alerts, and the labelling of molecules as selective inhibitors despite insufficient potency, an incomplete understanding of the structure–activity relationships (SARs) or lack of selectivity profiling. We conclude by offering thoughts on how this field may progress in the future.

## 2 Small GTPase Regulatory Proteins

### 2.1 GEFs

Each GTPase subfamily is associated with its own family of GEFs. The Rho and Arf GTPases each have two distinct GEF families, Dbl‐homology (DH) and Dock homology region (DHR) and Sec7 and Sec12, respectively. The size of the GEF families differs between the GTPase subfamilies; the Ras GTPase subfamily is larger than its GEF family, with 36 compared to 27 members, whereas the Rho GEFs outnumber the Rho GTPases three to one, allowing a more rigorous spatial control of activity.

**Figure 3:**
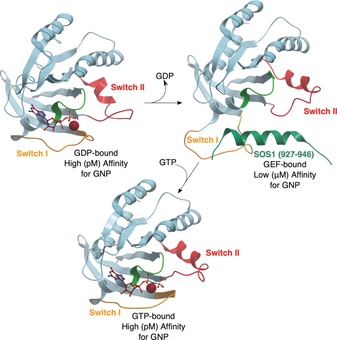
Structures showing conformational changes during nucleotide exchange. Switch I shown in orange, switch II in red, P‐loop in light green, GDP/GTP as purple sticks. A) PDB 4Q21. Ras bound to GDP. The switch II region is relatively disordered. B) PDB 1NVU. Nucleotide‐free Ras bound to Sos1 (interacting residues shown in dark green). The switch I loop has moved significantly and the switch II loop is more ordered. C) PDB 3L8Z. Ras bound to GTP analogue GPPNHP. The switch I loop has moved back but the switch II remains more helical.^5^

Although the general mechanism for GEF‐binding and the exchange reaction differs between subfamilies, some overarching similarities can be identified. The GEF associates with the GDP‐bound GTPase to form a low‐affinity complex. Upon nucleotide dissociation, this converts into a high‐affinity nucleotide‐free GTPase/GEF complex. Binding of GTP converts the complex back into a low‐affinity state and eventually the GEF is released, generating the active form of the GTPase. The GTP‐bound form is favored over the GDP bound form owing to the ten‐fold higher concentration of GTP in the cell. The GEFs bind to the GTPase, induce conformational changes within the switch I loop, and stabilize the complex by interacting with the switch II region (Figure 3).5b The GEFs generate the nucleotide‐free complex either by inserting residues to hinder the GDP phosphate‐binding region and/or the Mg^2+^, or remodeling switch II to destabilize the guanine nucleotide.^4^, 5b Compared to the intrinsic rate of reaction, GEFs can increase the rate of exchange by several orders of magnitude.^4^

### 2.2 GAPs

As with the GEFs, each subfamily has its own structurally distinct GAPs, although they are not as well characterized as their GEF counterparts. There is a similar pattern with the number of family members; Ras (14 GAPs)^16^ versus the larger Rho family (>66 GAPs),^17^ to Ran GTPase only having one, RanGAP.

**Figure 4:**
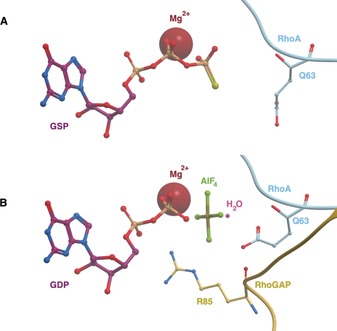
Ras, Rho, and Arf subfamilies’ GAP mechanism. A) PDB 1FTN. In RhoA‐GTP, the key catalytic glutamine (Q63, RhoA nomenclature) is oriented away from the nucleotide. B) PDB ITX4. Upon RhoGAP binding, is Q63 oriented towards a water molecule ready to catalyze hydrolysis of GTP. The arginine finger (R85, RhoGAP nomenclature) helps to stabilize the negative charge of the transition state (modelled as GDP and ALF_4_).

To prevent constitutive activity, the small GTPases have their own low intrinsic hydrolysis activity. The GAPs accelerate the hydrolysis of GTP to GDP by several orders of magnitude to enable the GTPase to be switched off rapidly if needed.5a As with the GEFs, the precise mechanism of this interaction between the GAP and GTPase is dependent on the GAP subfamily. The best understood mechanism (for Ras, Rho, and Arf GAPs, shown in Figure 4) involves the use of an arginine finger found on the GAP. This arginine finger orientates a conserved glutamine residue on the switch II region of the GTPase to activate a water molecule for nucleophilic attack of the GTP.8b, 18 The arginine and glutamine residues also stabilize negative charges found in the transition state. In the Rab subfamily, both the arginine and glutamine residues are provided by the RanGAPs. Ran GTPase provides the necessary arginine and tyrosine (in switch I) residues needed for GTP hydrolysis whilst RanGAP stabilizes the transition state.5b

### 2.3 GDIs

GDIs are only found for the Rho and Rab GTPase subfamilies, meaning a strategy to target GTPases through GDIs would not be applicable to the entire superfamily. Each family of GDIs contain only three members, with few isoforms. Three mechanisms have been identified for GDIs: 1) They bind to GDP‐bound forms of GTPases and act as a negative regulator by preventing nucleotide exchange, 2) they bind to the active GTP‐bound form and prevent intrinsic and GAP‐activated hydrolysis, thereby maintaining the GTPase in the active form, and 3) they are responsible for membrane release.8c

### 2.4 Targeting Strategies

Most of the inhibition strategies highlighted in this review target the GEFs (see Table 1). Their roles and mechanisms in the cell are better characterized, and inhibition of GEF activity provides an obvious advantage; by inhibiting GEF activation of the GTPase, the active GTPase concentration in the cell would decrease owing to intrinsic hydrolysis. As diseases often arise from faulty activation or overexpression of the GTPase, inhibition of the activation would be of therapeutic benefit. In this review, we have highlighted several methods of GEF inhibition, inhibition of the protein–protein interactions between the GTPase and GEF, targeting the GTPase/GEF complex, and inhibition of GEF activation.

**Table 1:** GEFs, GAPs, and GDIs mentioned in this review. Most research on identifying small molecule therapeutics has been concentrated on the Ras, Rho, and Arf GEF families.

GTPase subfamily	GEF subfamily	GEFs	GAPs	GDIs
Ras	Cdc25	Sos, Epac1/2, RasGRF1	NF1‐GAP	N/A^[a]^
				
Rho	Dbl	Trio, GEF‐H1, Vav2, ITSN, Tiam1, LARG, Dbs, PDZ‐RhoGEF	MgcRacGAP	RhoGDI1
	DOCK	DOCK1, DOCK2, DOCK5		
				
Rab		NI^[b]^	NI^[b]^	NI^[b]^
				
Ran		NI^[b]^	NI^[b]^	N/A^[a]^
				
Arf	Sec7	CYTH1, ARNO, BIG1, GBF1	ArfGAP1	N/A^[a]^
	Sec12	NI^[b]^

[a] This subfamily does not have GDIs. [b] No inhibitors in this review

**Figure 5:**
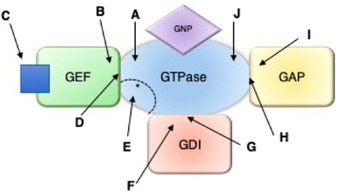
Methods of targeting GEFs, GAPs and GDIs. A) Binding to GTPase and blocking GEF binding. B) Binding to GEF and blocking GTPase binding. C) Preventing GEF activation. D) Binding to the GEF/GTPase complex. E) Targeting the effector binding site on the GTPase that overlaps with the GEF binding site. F) Binding to the GDI and blocking GTPase binding. G) Binding to the GDI/GTPase complex. H) Binding to the GAP/GTPase complex. I) Binding to the GAP. J) Binding to the GTPase to mimic GAP. Relevant references for each example are shown in Table 2.

Fewer molecules target the GAPs and GDIs. The design of a therapeutic strategy for the GAPs is more complicated than for the GEFs. On the one hand, finding a small molecule or biologic mimic that can promote hydrolysis on the GTPase would be beneficial in targeting constitutively active Ras. However, in the case of Ras mutants, this strategy has been as of yet unsuccessful, as mutations that prevent the GAP from binding to the GTPase also occlude small molecules from the key residues involved in GTP hydrolysis.^19^ On the other hand, inhibitors of GAPs have been published and may have some therapeutic value. At the very least, inhibitors of GAP activity could be useful as probes in the cell. The inhibition of GDIs can only be used to target the Rho and Rab subfamilies. We hypothesize that either compounds that stabilize the GDI–GTPase inactive complex, or inhibitors of the active GTPase/GDI complex would be of therapeutic benefit depending upon the specific GDI, GTPase, and disease. However, very little has been published regarding small molecule or peptide binders of the GDIs, perhaps owing to a relative lack of knowledge on their mechanisms in comparison to the body of knowledge published on GEFs. All of these strategies are highlighted in Figure 5 and Table 2.

**Table 2:** Compounds (given by their numbers) and biologics (by their names) listed in the review are characterized by their mechanism and GTPase target. There are no compounds targeting the Rab or Arf subfamiles.

	A	B	C	D	E	F	G	H	I	J
Ras	**1**–**13** HBS3 SAH‐SOS1_A_ KRpep‐2d	**14**–**21**	**22**–**27**	**28**–**33**	**34**–**37**					NF1‐S
Rho	**38**–**48**	**49**–**55**					**56**	**57**		
Arf	**58**	**62**–**70**		**59**–**61**					**71**

## 3 Ras GTPases

### 3.1 Small Molecule Ras Inhibitors (Method A)

Small molecules and peptides have been designed to bind to the GEF binding site of the GTPase, inhibit GEF/GTPase protein–protein interactions (PPIs), and so prevent formation of the complex and GDP turnover. For the examples detailed in this section, it has been reported that they either sterically block the GEF interaction with Ras or bind to Ras and lock it in a conformational state that is unfavorable for GEF binding. However, based on the structural data available, these mechanisms of action appear to be indistinguishable, and so the compounds in this mechanistic class are grouped below.

**Figure 6:**
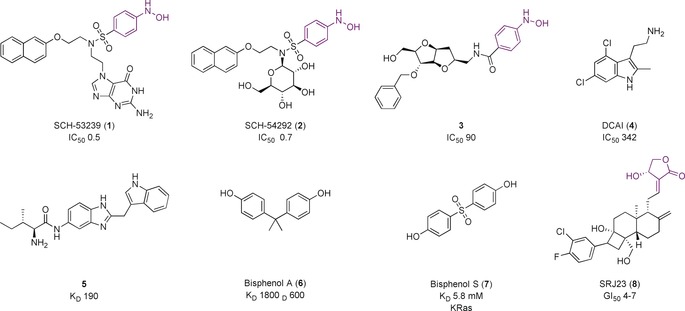
Compounds that block RasGEF–Sos interaction with Ras GTPase. Toxicophores/structural alerts are shown in purple.

SCH‐53239 (**1,** Figure 6) was designed by the Schering‐Plough Institute and intended to deactivate Ras by binding competitively with GDP. However, a model of SCH‐54942 (**2**), derived from NMR spectroscopy experiments, determined that the compounds bound to a major hydrophobic cleft in the switch II region of Ras.^20^ Unfortunately, despite their potency, the compounds had low solubility in aqueous or organic solvents and were relatively unstable, hindering the development of these compounds as drugs or probes. Nevertheless, they inspired the generation of compounds that contain two aromatic pharmacophores linked through a spacer, but with improved water solubility. The most potent series contained an arabinose‐derived bicyclic linker, such as **3**, which had a mild cytotoxic effect in cells expressing oncogenic Ras and an IC_50_ value of 90 μm against Sos‐mediated nucleotide exchange.^21^ The binding site of the series was confirmed to overlap with the large binding interface of Sos. It is therefore supposed that these molecules inhibit nucleotide exchange by sterically blocking GEF‐binding to the Ras GTPase. However, the entire series contains an aromatic hydroxylamine motif, which is commonly associated with toxicity problems in potential therapeutics, although this may not be an issue for a chemical probe.^22^

Two groups, from Genentech and Vanderbilt University, found hits that bound in the same novel pocket of KRas adjacent to the switch I/II region, but distinct from the binding site of the Schering‐Plough compounds. Maurer et al.^23^ used a saturation transfer difference (STD) NMR assay to screen 3300 fragments. Validation using ^1^H–^15^N HSQC NMR spectroscopy identified 25 compounds of interest. Co‐crystal structures of benzamidine, benzimidazole, and 4,6‐dichloro‐2‐methyl‐3‐aminoethyl‐indole (DCAI) (**4**, Figure 6) showed binding within a hydrophobic pocket situated between the central β sheet of the protein and the switch II helix. **4** bound to KRas with weak affinity (*K*_D_=1.1 mm), and inhibited Sos‐catalyzed nucleotide exchange with an IC_50_ value of 342 μm. Several biochemical studies confirmed that **4** disrupts nucleotide exchange by sterically blocking the formation of the Ras‐Sos complex. In cells, **4** disrupted Ras activity with an EC_50_ value of 16 μm, much lower than expected considering its binding potency, and the authors do not rule out off‐target mechanisms.

Sun et al.^24^ used NMR screening of 11 000 fragments to yield 140 hits that bound to KRas with 1.3–2 mm affinity. Co‐crystal structures for 20 of the compounds indicated they bound in the same pocket as the compounds discovered by Maurer et al. A series of indole analogues were synthesized, guided by interactions identified in the crystal structures, and improved the affinity to a *K*_D_ value of 190 μm for **5** (Figure 6), which achieved 78 % inhibition of Sos‐mediated catalyzed exchange with 1 mm concentration in vitro. These compounds, whilst located in the same binding pocket as before, also extended into a secondary binding site nearby. The authors summarize that these compounds would be good starting points for a probe and have so far been used to develop covalent “tethering” compounds, which saturate the binding site to allow fragment screening of a second pocket.^25^ The fragments from the second screen bound to KRas with affinities of 0.3–3 mm and could be linked to the original indoles to develop more potent inhibitors, although no follow‐up compounds have currently been reported.

Inspired by these results, Schöpel et al.^26^ designed a fragment library of 100 compounds based on SAR data from known Ras inhibitors, and used multidimensional NMR spectroscopy to identify molecules that bind to Ras homologue enriched in the brain (Rheb). Compounds were screened against KRas for selectivity. Bisphenol A (**6**, Figure 6) bound to Rheb and KRas with *K*_D_ values of 1800 and 600 μm, respectively. NMR data and in silico models show the binding site to be the same as for **4** and **5**. Further studies tested the analogue bisphenol S (**7**) on KRas to see if it mimicked the binding of BPA.^27^ However, **7** had a *K*_D_ value of 5.8 mm and no effect on Sos‐mediated exchange. Docking results suggested that the sulfone group of **7** is too bulky to fit into the binding pocket.

Molecular dynamics (MD) studies have predicted four allosteric binding sites on KRas, which indicate non‐rigid conformations exist that could be targeted with small molecules.^28^ As confirmation of the method, the pocket targeted by **4**, **5**, **6**, and **7** was predicted in this analysis.^29^ The ensemble models were used to analyze whether the anticancer effect of the natural product andrographolide (AGP), and derivatives such as SRJ23 (**8,** Figure 6), were directly attributable to inhibition of Ras.^30^ Mapping of these compounds found their preferred hotspot differed to the binding sites of **4** and **5. 8** was predicted to displace residues involved in Sos binding and stabilize the “open” conformer of switch I in a state that is non‐conducive to GEF binding. **8** reduced K‐, H‐, and NRas‐GTP levels in cells at concentrations of 1–7.5 μm when incubated for 6 h, and induced growth inhibition in three different cancer cell lines (GI_50_=4–7 μm). However, it is difficult to attribute this effect to selective inhibition of Ras without in vitro potency data.

**Figure 7:**
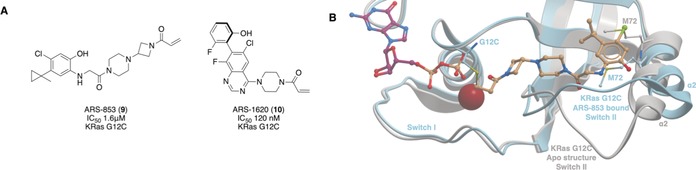
A) Covalent inhibitors of KRas G12C. B) Binding mode of **9** to KRas G12C (blue, PDB 5F2E) compared to the apo KRas (grey, PDB 4OBE). The binding of **9** caused the α2 helix and Met72 of switch II to move. The carbonyl of the acrylamide is situated where the γ‐phosphate of GTP would be situated. These changes result in the GDP‐state of KRas being favored and prevent nucleotide exchange.

**Figure 8:**
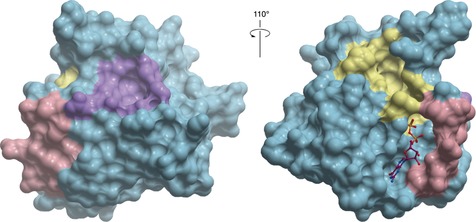
Binding interactions of the Ras‐GEF PPI inhibitors. The purple surface corresponds to the binding site of **4**, **5**, **6**, and **7**. Pink corresponds to the residues interacting with **8**. Yellow corresponds to the residues interacting with **1**, **2**, **3**, **9**, and **10**. The GDP binding site is shown with the GDP as purple sticks, PDB 4EPY.^35^

The mutant G12C Ras is hyper‐active in cancer, and theoretically could be specifically targeted with cysteine‐reactive small molecules over wild‐type Ras. Using fragment‐based screening and structure‐guided design, Ostrem et al.^31^ identified a series of inhibitors that covalently bound to C12 in an allosteric pocket beneath switch II, causing conformational change of residues in switch I. The compounds preferentially bound to Ras‐GDP, impaired the function of Sos, and blocked nucleotide exchange. ARS‐853 (**9**, Figure 7 A) was designed after the original compounds did not have substantial KRas G12C engagement in cells.^32^ **9** had a cellular engagement IC_50_ value of 1.6 μm after 6 h; mutation studies and structural analysis showed that **9** trapped KRas G12C in the inactive state by lowering its affinity for GEFs and thereby attenuating Sos‐mediated nucleotide‐exchange (Figure 7 B).^33^ Poor pharmacokinetic properties meant **9** was unsuitable for in vivo studies. Optimization yielded ARS‐1620 (**10**), which had an IC_50_ value of 120 nm in cells and a proteomic screen established that KRas G12C was the most substantially labelled cysteine residue in the proteome.^34^ It also had improved pharmacokinetic properties in vivo. **10** significantly and selectively inhibited tumor growth in xenograft mouse models containing the KRas G12C mutation in comparison to a negative control or a tumor harboring KRas G12V. In a panel of patient‐derived models, **10** achieved high target occupancy of G12C and inhibited phosphorylation downstream of Ras with no signs of toxicity in mice. Hence **10** can be used as a probe, both in vitro and in vivo to investigate KRas G12C inhibition. Structures showing the binding sites for all small molecule inhibitors of Ras are shown in Figure 8.^35^

**Figure 9:**
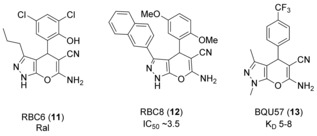
Inhibitors of Ral‐GDP.

The Ral (Ras‐like) proteins, a subset of the Ras subfamily, have emerged as critical targets in cancer therapy.^36^ 500 000 compounds were screened in silico in a pocket identified in RalA‐GDP that is not present in the RalA or B GNP structure.^37^ It was anticipated that compounds binding in this pocket would prevent the activation of the Ral GTPases by their GEFs. 88 compounds were tested in two cell‐based assays; RBC6 and RBC8 (**11** and **12**, Figure 9) were chosen for their ability to reduce RalA activation in cells, and binding in the desired pocket was confirmed by NMR spectroscopy. Synthesis of derivatives based on the bicyclic scaffold yielded BQU57 (**13**) as a more potent binder (*K*_D_=5–8 μm by ITC and SPR) with superior drug‐like properties. Application of **13** in human lung cancer cell lines showed selective inhibition of Ral activity in Ral‐dependent cell types with an IC_50_ value of 1–2 μm. **12** and **13** were tested in vivo, although their low micromolar potency means their antitumor properties may be in part due to off‐target binding.

### 3.2 Peptide Ras Inhibitors (Method A)

Peptide mimetics have also been developed to inhibit Ras‐GEF interactions. Structural analyses identified a helical hairpin in Sos key to disrupting interactions between Ras and the guanine nucleotide. Patgiri et al.^38^ hypothesized that mimetics of the αH helix of the hairpin (residues 929–944), the only structural part of the hairpin to directly interact with Ras, could compete with Sos for Ras binding. Synthetic mimics were generated in which the native sequence was optimized to improve solubility and a hydrogen‐bond surrogate (HBS) approach stabilized the helix. HBS3 had a *K*_D_ value for nucleotide‐free Ras of 28 μm and GDP‐bound Ras of 158 μm, whereas Sos has a *K*_D_ value of 14.5 μm for GDP‐bound Ras. HBS3 permeated the cell membrane and reduced Ras activation in cells due to direct inhibition of the Ras–Sos interaction.

Leshchiner et al.^39^ generated a series of stapled α helices (SAH‐SOS) that again replicated the secondary structure of Sos (929–944). The lead peptide, named SAH‐SOS1_*A*_, was found by a fluorescence polarization (FP) assay to bind to KRas WT and common mutant forms with EC_50_ values in a range of 100–175 nm. SAH‐SOS1_*A*_ showed dose‐dependent inhibition of nucleotide association, with a negative control, SAH‐SOS_*B*_, having no effect in a nucleotide exchange assay. In KRas mutant cancer cells, SAH‐SOS1_*A*_ impaired cell viability in a manner dependent on KRas inhibition. They concluded that the optimization of peptides using SAR and further cellular studies is required, and a patent has been obtained,^40^ indicating the promise of this method for Ras–Sos inhibition.

Sacco et al.^41^ developed peptides derived from the sequence of dominant negative mutants of RasGRF1 (a different RasGEF) that inhibited Ras both in vitro and in vivo. Trp 1056 was identified as a key residue; mutating Trp 1056 to Glu maintained Ras specificity and affinity for GTP but was catalytically inactive. A peptide of 67 residues centered on Trp 1056 was designed. This peptide, and the Tat‐fused truncated analogue designed to improve mammalian cell penetration down‐regulated Ras activity in cells, and the latter inhibited in vitro GEF‐mediated nucleotide exchange. However, the potency was not quantitatively determined, nor off‐target effects explored prior to cellular studies, meaning it is difficult to definitively attribute cell phenotypes to Ras inhibition through the arresting of nucleotide exchange.

A random peptide library displayed on T7 phage was screened against KRas‐G12D identifying 3 consensus sequences. Subsequent evaluation by surface plasmon resonance (SPR) and enhancements led to KRpep‐2d, which was stabilized by an intramolecular disulfide bridge and inhibited Sos‐catalyzed nucleotide‐exchange with an IC_50_ value of 1.6 nm.^42^ KRpep‐2d inhibited cancer cell proliferation at 30 μm, which is high compared to the in vitro IC_50_ value. The crystal structure of KRas G12D in complex with GDP and KRpep‐2d was obtained, confirming that KRpep‐2d bound to a cleft near the switch II region.^43^ KRpep‐2d acts as an allosteric inhibitor, stabilizing the switch II region (distal to Sos‐binding site) in a conformation non‐conducive to nucleotide exchange. However, it is hypothesized that some of the inhibitory effects seen with KRpep‐2d could be due to the effect of the switch II conformational change on Ras–effector binding as well as Ras–GEF interactions.

### 3.3 RasGEF Inhibitors (Method B)

Evelyn and colleagues reported direct inhibitors of Sos1 in two different screens.^44^ In the first, crystal structures of the Ras–Sos complex were used in a virtual screen to enable rational design of compounds that inhibit formation of the complex.44a

**Figure 10:**
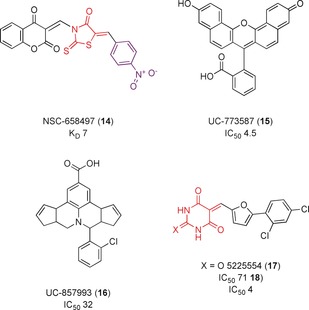
RasGEF inhibitors. PAINs motifs are shown in red and toxicophores/structural alerts in purple.

From 18 500 small molecules, 36 were chosen for experimental validation. The most active compound, NSC‐658497 (**14**, Figure 10), completely inhibited the Sos1‐catalyzed exchange reaction on HRas at 100 μm and bound to Sos1 with a *K*_D_ value of 7 μm but not HRas. Alanine scanning mutagenesis studies showed that **14** bound to the Sos1 catalytic site involved with interactions with the HRas switch II region. However, the rhodanine moiety has been identified as a PAINs motif for reactivity and chelation.^45^ Hence, further target validation studies are required to ensure Ras inhibition seen in cells are definitively due to the binding to Sos1.

In the second screen, they identified two compounds, UC‐773587 and UC‐857993 (**15** and **16**, Figure 10), which bound selectively to the catalytic site of Sos1 over HRas and ITSN (a RhoGEF) and inhibited nucleotide exchange with IC_50_ values of 4.5 and 32 μm, respectively.44b Alanine scanning mutagenesis indicated that **15** mapped to the Ras switch II interaction region of the Sos1 catalytic site and **16** to the Ras switch I interaction region. Furthermore, **15** and **16** additively inhibited growth of Sos1‐dependent DU‐145 prostate cancer cells, showing that these compounds can act additively in suppressing Sos1 activity. However, both of these compounds are highly colored and are likely to interfere in assays.

Sampling of potential druggable sites on Epac (Rap GEF modulated by cAMP, isoforms Epac1 and Epac2) identified the hinge region, which bends upon cAMP binding to the cyclic‐nucleotide binding domain (CNBD), resulting in conformational changes that activates Epac.^46^ Four compounds identified from virtual screening and a BRET‐based assay decreased Epac1 activation. Further characterization showed that only one, the barbituate 5225554 (**17**, Figure 10), inhibited Rap1 activation in cells. The thiobarbituric acid derivative 5376753 (**18**) was identified as the more potent Epac1 inhibitor in Swiss 3T3 cells, with an IC_50_ value of 4 μm. **17** and **18** were hypothesized to prevent the conformational change of Epac necessary for its activation, resulting in an inhibition of its ability to act as a GEF. **17** was determined to be unsuitable, owing to its low solubility and toxicity effects, but it was suggested that further development of **18** could result in a more potent inhibitor. However, the barbiturate group has been identified as a PAINs motif owing to its high reactivity.^45^

Further studies should be conducted to ensure there are no off‐target effects before use in cells. Despite this, and the recommendation of the authors that further SAR studies should be conducted to generate compounds with greater potency, **18** is being advertised as an allosteric inhibitor that can be used to study the effect of Epac inhibition in cells.^47^

**Figure 11:**
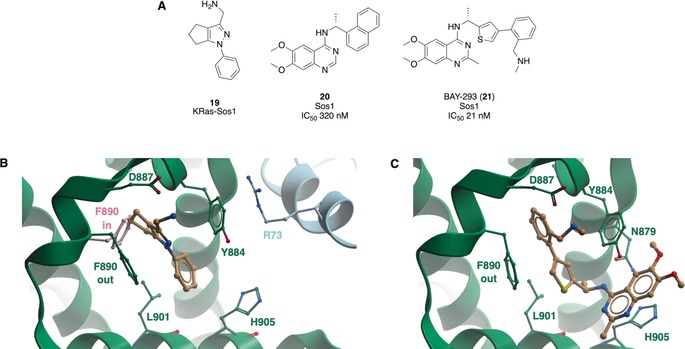
A) Compounds discovered to bind to Sos1. B) **19** bound to a pocket in Sos1 (green) adjacent to KRas (blue) (PDB 6EPM). It formed hydrogen bonds with Tyr884 and Asp887 and forced a Phe890‐out conformation in comparison to the Phe890‐in conformation seen in the apo structure (pink, PDB 6EPL). C) **21** bound to Sos1 (green, PDB 5OVI), maintaining the Phe‐out conformation of **19** but expanding into the neighbouring pocket where **20** is found to bind. Key interacting residues are labelled.

Hillig et al. at Bayer recently developed a potent compound that inhibited the KRas^G12C^‐Sos interaction with an IC_50_ value of 21 nm.^48^ A STD NMR screen of 3000 fragments to find stabilizers of the complex identified 97 fragments that bound to KRas–Sos1. Co‐crystal structures showed that the fragments bound a hydrophobic pocket on Sos1. Some of these fragments, including **19** (Figure 11 A), induced a rotation of Phe890 to open a new back pocket (Figure 11 B) and stabilised the KRas–So1s complex in 2D NMR spectroscopy, SPR assay, and a KRas–Sos1 biochemical interaction assay. However, analogues based on **19** did not result in an improvement in potency.

A parallel HTS screen of over 3 000 000 compounds using a fluorescent enzymatic assay identified a quinazoline series, such as **20**, which inhibited the activation of KRas by Sos1 with submicromolar affinity and was selective over other GEF/GTPase complexes and the interaction of KRas with its effector. Thermal shift assays, ITC, and native mass spectrometry established that **20** bound directly to Sos1 with a *K*_D_ value of 450 nm and disrupted the KRas–Sos1 interaction. Co‐crystal structures of **20** revealed a binding pocket on the surface of Sos1, with the napthyl group accessing the same pocket as the fragments. Further SAR studies showed selectivity for Sos1 over its close homolog Sos2, other RasGEFs and a kinase panel, although the potency could not be improved beyond 130 nm. To combine access to the fragment‐induced sub‐pocket with the potency of the HTS series, it was decided to merge the two series. Linkers were optimised computationally, with a thiophene chosen for potency and ease of synthesis. Structural‐based design and exploration of SARs yielded a racemate of which the (*R*)‐enantiomer (BAY‐293, **21**) had an IC_50_ value of 21 nm, and was shown to bind to both pockets of **19** and **20** (Figure 11 C). Cellular studies showed that the **21** inhibited Ras activation in HeLa cells with submicromolar IC_50_ values in comparison to the (*S*)‐enantiomer as a negative control. In cell lines containing the KRasG12C mutation, which are thought to be less dependent on their exchange factors, pERK activity was still reduced by 50 %. This downstream effect was improved by the combination of **9** with **21**, which showed synergistic antiproliferative activity in KRasG12C mutant cells, and highlights the opportunity for combination therapy with KRas G12C covalent and Sos1 direct inhibitors. The bioavailability of **21** needs to be improved prior to use in in vivo experiments, although it is suitable as a probe in vitro to study the effects of Sos1 inhibition.

### 3.4 Inhibition of RasGEF Regulation (Method C)

Some GEFs can only catalyze nucleotide exchange when bound to binding partners. The Epac RasGEFs are directly activated through the binding of cAMP.^49^ When cAMP is not bound, the Epac proteins are auto‐inhibited. Dock‐A and Dock‐B, subfamilies of the DOCK family of Rho GEFs, must form a bipartite complex with a member of the ELMO family of adaptor proteins before activation of the GTPases can be achieved.^50^ It is theoretically possible that drugs targeting these regulatory molecules/proteins would prevent activation of the GEF and inhibit nucleotide exchange, although cAMP/Epac interaction inhibitors are the only reported examples.

**Figure 12:**
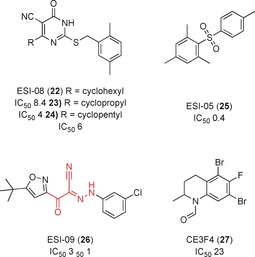
Inhibitors of Epac activation by blocking the interaction with regulatory cAMP. PAINs motifs highlighted in red.

A series of novel cAMP competitive inhibitors were identified using a high‐throughput fluorescence competition assay, in which molecules displaced a fluorescent analogue of cAMP.^51^ Screening of 1990 diverse molecules yielded three compounds that had IC_50_ values between 1–8 μm. No further modification of these compounds has been published following this pilot screen. Following this validation of the assay, 14 400 drug‐like molecules were screened; seven molecules inhibited Epac2 catalyzed nucleotide exchange at 25 μm.^52^ SAR studies for ESI‐08 (**22**, Figure 12) were explored by Chen et al.^53^ through the synthesis of a series of 5‐cyano‐6‐oxo‐1,6‐dihydro‐pyrimidine derivatives. Modifications at the C‐6 position of the pyrimidine ring improved specificity for Epac2 over Epac1, and docking predicted the binding of these compounds to the CNBD of Epac1. The authors identified two of these compounds, HJC0198 and HJC0197 (**23** and **24**) as “pharmacological probes”, despite the most potent, **23**, having an IC_50_ value of only 4 μm in vitro.

The sulfone ESI‐05, (**25**, Figure 12) was selective for the Epac2 isoform over Epac1, with an IC_50_ value of 0.4 μm. cAMP had an IC_50_ value of 40 μm in the same assay, establishing **25** as a potent inhibitor in comparison to the native substrate. **25** showed Epac2‐dependent reduction of Rap1 activation in HEK293 cells. It was hypothesized that the specificity for the single Epac isoform was due to **25** binding along the interface of two CNBDs in Epac2, compared to Epac1, which has only one CNBD. Further SAR studies yielded a structural analogue with an IC_50_ value of 0.3 μm in competitive binding to Epac2, representing a 133‐fold greater potency over cAMP.^54^ Molecular docking of the ligands at the CNBD‐A and CNBD‐B interface provided additional weight to the hypothesis that the compounds bound at this allosteric site.^55^

ESI‐09 (**26**, Figure 12) inhibited cAMP‐mediated Epac1 and 2 GEF activities with IC_50_ values of 11 μm and 4.4 μm, respectively.^56^ Molecular docking predicted binding to a single CNBD domain in both proteins.^57^ **26** was used to show that Epac proteins have a role in the migration of pancreatic cancer cells, and in vivo significantly reduced pancreatic cancer cell invasion and metathesis.^57^, ^58^

However, it was suggested that **26** has general protein denaturing qualities instead of direct inhibition of Epac.^59^ The cyano‐imine and imine‐ketone groups have also both been identified as PAINs motifs.45a The authors believe the docking results, in vitro and in vivo data in which **26** can recapitulate Epac1 knockout phenotypes,^60^ and SAR regarding the importance of chloro‐substituents on the phenyl ring are sufficient to validate it as an Epac‐specific antagonist and not simply a PAIN. Further structural modifications led to compounds with improved solubilities and sub‐micromolar and micromolar IC_50_ values for Epac2 and Epac1, respectively.^61^

The ESI compounds are examples of competitive cAMP inhibitors; an uncompetitive inhibitor of Epac1, CE3F4 (**27**, Figure 12), was identified by Courilleau et al.^62^ Using a high‐throughput fluorescent assay, 640 compounds from the French National Chemical Library were screened for their ability to inhibit Epac1 activity. The tetrahydroquinoline analogue **27** inhibited the cAMP induced exchange activity of Epac1 with an IC_50_ value of 23 μm. **27** inhibited Epac1 but not Rap1 or the Rap1–Epac1 interaction. Further studies showed that **27** prevented conformational change induced by agonist binding, necessary for relieving the autoinhibitory mechanisms that prevent Epac1 from activating Rap1. Further investigation showed that the (*R*)‐**27** enantiomer has greater potency than racemic or (*S*)‐**27**, with an IC_50_ value against Epac GEF activity of 4.2 μm.^63^ Recent SAR studies on tetrahydroquinoline analogues^64^ indicated that the two bromine atoms and formyl group are essential for activity. A patent for the use of tetrahydroquinolines as Epac1 inhibitors has been filed.^65^

### 3.5 Binders of the Ras/RasGEF Complex (Method D)

AstraZeneca^66^ intended to develop a compound to bind to the KRas/Sos interface and stabilize the complex, thereby inhibiting dissociation of Sos and preventing activation of Ras. A screen using X‐ray crystallography found fragments that bound to Sos on the interface of HRas (used as no reported structure for KRas) and Sos. Despite several rounds of chemical optimization to improve potency, they were unable to demonstrate that the fragments could stabilize the complex. It was decided to pursue a covalent molecule to irreversibly inhibit the protein. They identified Cys118R as being proximal to the GDP binding site and screened 400 compounds using mass spectrometry. Structure‐based design resulted in a series of N‐substituted maleimides (Table 3) that once bound partly occluded the nucleotide binding site. Complete inhibition was only achieved when incubated with KRas‐GDP/Sos complex rather than KRas‐GDP alone. It is thought these compounds inhibit nucleotide exchange by locking the complex in an abortive state, as the loop containing Cys118R is unable to move back to the conformational state that can bind to nucleotides. However, the cysteine selectivity of these compounds is unknown and further work is required to optimize the covalent warhead.

**Table 3:** Representative structures of N‐substituted maleimides targeting Cys188R of KRas.

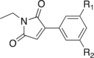	R	R_2_
**28**	CH_2_NH_2_	H
**29**	CONH_2_	H
**30**	COOH	CN
**31**	CONH_2_	Cl

**Figure 13:**
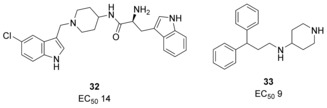
Compounds that activate Sos‐catalyzed nucleotide exchange and induce biphasic responses in cells.

A series of aminopiperidine indoles discovered by Burns et al.^67^ bound to a hydrophobic pocket of the Cdc25 domain of Sos by NMR spectroscopy and increased the rate of Sos‐catalyzed nucleotide exchange in vitro; the most potent of these, **32** (Figure 13), had an EC_50_ value of 14 μm. X‐ray crystal structures showed the compounds bind in a hydrophobic pocket of Sos, with key interactions with the switch II of Ras, in the Ras/Sos/Ras complex. Mutation studies of Ras showed this pocket to be critical for Sos‐mediated nucleotide exchange. An additional HTS of over 160 000 compounds in a fluorescence‐based screening assay yielded 244 hits, of which six were prioritized for follow‐up analogues.^68^ X‐ray crystal structures confirmed the compounds bound in the same pocket as **32**. Interestingly, only **32** and one additional compound, **33**, were able to elicit biphasic responses in phospho‐ERK levels downstream of Ras in cells; at low concentrations an increase in ERK phosphorylation was seen, but high concentrations inhibited ERK phosphorylation. This was found to be related to the compounds that have a higher maximal rate of nucleotide exchange in cells, rather than their EC_50_. Further studies showed that a negative feedback mechanism is induced to override the activation of Ras‐GTP by a compound, resulting in an overall decrease in downstream ERK signaling.^69^ The authors conclude that the structurally diverse scaffolds are a starting point in the discovery of more potent molecules that act as inhibitors of signaling pathways downstream of Ras, if they activate Ras past the threshold that induces the negative feedback loop.

### 3.6 Modulation of GTPase–Effector Interactions (Method E)

**Figure 14:**
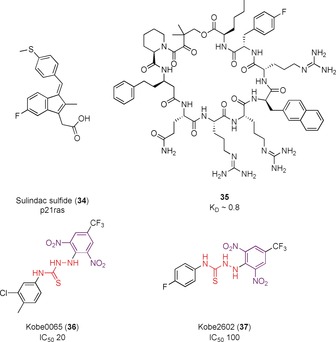
Inhibitors of Ras‐effectors interactions, in which regulator binding is also affected. PAINs motifs highlighted in red and toxicophores/structural alerts in purple.

Small molecules and peptides have been designed to block the interactions of effectors with Ras‐GTP, preventing the activation of downstream signaling pathways.^70^ Structural studies have shown that the binding region of regulators and effectors overlap, and so some therapeutics designed to inhibit effectors have also been reported to block GEF or GAP function, some of which are shown in Figure 14.^71^ We also identified PAINs motifs associated with toxicity and low potency compounds used in assays in this subset of inhibitors.45a, 45b, 72 Whilst we will not go into detail about these compounds, as most research concentrated on investigating the effector rather than regulator inhibition, it is worth highlighting the inhibitors of effectors can also inhibit Ras regulators, and vice versa. Whilst inhibitors of Ras–effector interactions are promising, the side effects of inhibiting regulator activity highlight the difficulty of developing direct selective inhibitors of the GTPase proteins.

### 3.7 Ras Inhibitors Prevent Interaction with RasGAP (Method J)

Clark et al.^73^ identified a consensus binding sequence of eight amino acids shared between a subset of Ras effectors, including the RasGAP NF1‐GAP. 10‐ or 11‐amino‐acid peptides from NF1‐GAP and Raf1 containing this consensus sequence, known as NF1‐S and Raf‐S, inhibited NF1‐GAP stimulated hydrolysis with IC_50_ values of 87 and 44 μm, respectively. It is hypothesized that these peptides bind to Ras and block the Ras‐NF1‐GAP interaction. Whilst these peptides inhibited Ras‐mediated activation in an oocyte lysate assay, this was attributed to prevention of Raf1 activation rather than GAP inhibition.

## 4 Rho GTPases

### 4.1 Direct Inhibition of the Rho GTPases (Method A)

**Figure 15:**
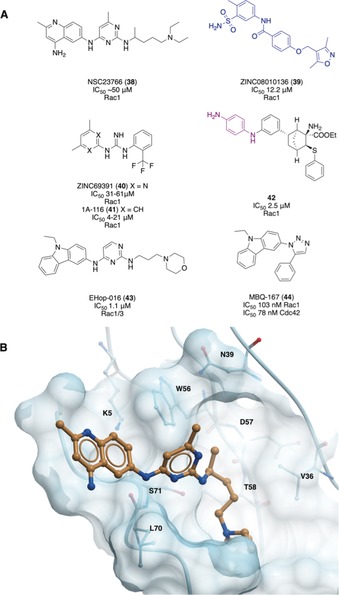
A) Elaboration of initial hit NSC23766 using structure guided design to yield potent derivatives. Structures similar to known aggregators are shown in blue. Toxicophores/structural alerts are shown in purple. B) Crystal structure of NSC23766 bound to Rac1. Important residues forming the pocket, including critical residue W56, are shown.^75^

Rho GTPases (20 members in the human genome)^2^ are outnumbered by their GEFs (>70 in the human genome);8d selective interaction by a subset of GEFs with a specific GTPase allows for control over the signaling pathways. Residues found mainly in the β1/β2 regions of the GTPase determine specificity for GEFs for three well‐studied Rho GTPases, Rac1, Cdc42, and RhoA. In Rac1, Trp 56 was identified as the critical residue for selectivity of GEFs. Introduction of Trp 56 into Cdc42, another member of the Rho subfamily, resulted in Cdc42 being responsive in vitro and in vivo to the Rac1‐specific GEFs Trio, GEF‐H1, and Tiam1.9b In Cdc42, specificity is determined by Phe56 and in RhoA, Trp 58. Gao et al.^74^ used these residue differences to discover a small‐molecule inhibitor specific to Rac1. Using a structure‐based virtual screening approach of over 140 000 molecules, in a putative inhibitor binding pocket targeting the key residues, they identified NSC23766 (**38**, Figure 15 A,B), which was selective for Rac1/TrioN over Cdc42/ITSN and RhoA/PDZ‐RhoGEF. It had IC_50_≈50 μm in vitro for the GEF‐catalyzed nucleotide exchange and showed a dose‐dependent inhibition of Rac1 activity in cellular assays, although this could be in part due to off‐target effects arising from the low potency.

This discovery inspired several virtual screening campaigns of the ZINC database using a pharmacophore model derived from the crystal structure of NSC23766 bound to Rac1 (Figure 15 B).^75^ Ferri et al.^76^ identified *N*‐(sulfamoylaryl)arylamide ZINC08010136 (**39**, Figure 15 A) as a Rac1‐selective inhibitor that could interfere with GEF/GTPase complex formation and Rac1 activity in cells (IC_50_=12 μm), although it has high structural similarity to a known aggregator.45a, 77 Further virtual screens explored the SARs and the most promising analogue had an IC_50_ value of 8.7 μm in cell‐based assays.^78^ Another campaign identified ZINC69391 (**40**), which blocked the Rac1–GEF interaction and Rac1 activity in cells, and was optimized to give 1A‐116 (**41**).^79^ **41** had an IC_50_ value of 4 μm for the antiproliferation of F3II cancer cells, was more potent in blocking GEF activation in vitro than **40**, and caused a 60 % reduction in metastatic lung colonies in vivo. A different approach used de novo design to yield an inhibitor that would have the same pharmacophoric features as **38** and other previous compounds but a unrelated scaffold.^80^ In cellular assays, **42** reduced Rac1‐GTP levels with an IC_50_ value of 2.5 μm albeit containing a dianiline structural alert motif. However, all of these compounds have similar scaffolds and have not dramatically improved potency.

Montalvo‐Ortiz et al. attempted to elaborate **38** to produce a compound with greater potency, although this led to a loss of selectivity.^81^ Optimization led to the identification of EHop‐016 (**43**, Figure 15 A). Molecular docking indicated **43** bound with deeper interactions into the same putative GEF binding pocket as **38**, but not in the same orientation. In the MDA‐MB‐435 cell line, **43** had an IC_50_ value of 1.1 μm whereas **38** had an IC_50_ value of 96 μm. However, above 5 μm, **43** inhibited Cdc42. Studies in cells indicated **43** reduced tumor growth, metastasis, and angiogenesis, with no apparent toxicity, indicating potential as a cancer therapeutic.^82^ Further modifications of the scaffold to bind deeper into the pocket yielded MBQ‐167 (**44**), which inhibited Rac1 activity in cells with an IC_50_ value of 103 nm.^83^ However, it also blocked Cdc42 activity with an IC_50_ value of 78 nm, meaning selectivity had been lost. It prevented mammary tumor progression in vivo 10‐fold more potently than **43**, although additional effects from an unknown alternate mechanism were also identified.

**Figure 16:**
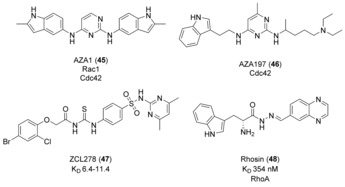
Small molecules developed for targeting Cdc42 and RhoA.

Inhibitors of Cdc42 and RhoA have also been designed. AZA1 (**45**, Figure 16) was discovered using an in vitro screen of small‐molecule inhibitors based on **38**. It inhibited Rac1 and Cdc42 activity in cells at low‐micromolar concentrations.^84^ The same screening method identified AZA197 (**46**), which specifically inhibited Cdc42‐Dbs (RhoGEF) interactions by 61 % in vitro, although no IC_50_ value was calculated.^85^ It blocked Cdc42‐dependent migration and altered the cell morphology of cancer cells, whilst also suppressing colon cancer growth in vivo. ZCL278 (**47**) was found by virtual screening.^86^ 197 000 compounds were docked into a 16 residue GEF‐binding pocket of Cdc42, identified from analysis of the Cdc42‐ITSN (RhoGEF) complex crystal structure. This pocket included the key Phe56 residue found in Cdc42. Two independent biophysical methods established a *K*_D_ value of 6.4–11.4 μm. **47** inhibited Cdc42 and not RhoA or Rac1‐mediated phenotypes, including microspike formation and Cdc42‐mediated neuronal branching.

4 000 000 compounds were computationally docked into the shallow pocket of RhoA surrounding Trp 58, in which Rhosin (**48**, Figure 16) was identified as binding with a *K*_D_ value of 354 nm.^87^ Although LARG (RhoGEF)‐catalyzed nucleotide exchange was inhibited and no precise value was calculated, it was stated to be weaker than the *K*_D_ value. Mutation studies of key residues supported that **48** bound in the predicted pocket, blocking GEF‐catalyzed nucleotide exchange on RhoA. However, **48** also inhibited RhoB and RhoC activities in cells as they share identical surface residues in this binding site. It inhibited RhoA‐mediated cell function whilst not affecting Cdc42 or Rac1 phenotypes or showing cytotoxicity.

There have also been biologics that target the Rho GTPases and prevent their binding to GEFs. Contini et al. designed a peptide based on the sequence of Tiam1 to inhibit the protein–protein interaction between Tiam1 and Rac1.^88^ Simulations identified the CR3 helix of Tiam1 to be of principal importance in the Tiam1–Rac1 interaction, with stapling used to stabilize the peptide. The peptides synthesized were able to cross the cell membrane, overcoming a major issue with biologic therapeutics, but IC_50_ values could not be calculated in vitro, as even at 100 μm, 50 % inhibition had not been achieved. However, the reduction in activity was greater than **38**, which was used as a control in the assay.

### 4.2 RhoGEF Inhibitors (Method B)

**Figure 17:**
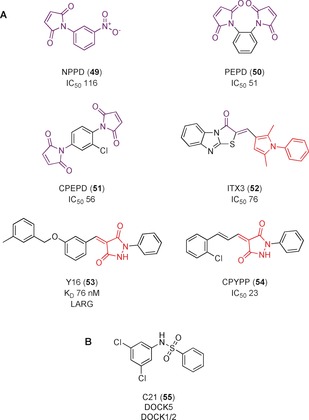
A) RhoGEF inhibitors that are highly reactive or contain a PAIN motif (red). Toxicophores of reactive motifs are shown in purple. B) C21, inhibitor of DOCK5, a DHR RhoGEF.

A yeast exchange assay was used to identify small‐molecule inhibitors of the RhoGEF Trio, which activates RhoG.9a 3 500 compounds were screened, with NPPD (**49**, Figure 17 A) identified as a potential Trio inhibitor (IC_50_=116 μm). 23 structural analogues were tested to improve potency and establish SAR; PEPD and CPEPD (**50** and **51**) were identified, with IC_50_ values of 51 and 56 μm, respectively, for the activity of Trio GEF against RhoG. However, further studies showed that **49** is toxic in mammalian cells, killing up to 90 % of cells after 48 h at 100 μm. This is not surprising, owing to the highly reactive nature of maleimides towards exposed cysteine residues.^89^ The initial screening compounds were reanalyzed and ITX1, structurally distinct from **49**, was chosen for its absence of toxicity whilst maintaining Trio inhibitory activity in vitro.^90^ A secondary screen of ITX1 analogues established ITX3, (**52**) as the most potent with an IC_50_ value of 76 μm. However, this subset of analogues contains a PAINs 1,2,3‐alkyl pyrrole motif,45a, 45b and this, combined with their mid‐micromolar potency, brings into question the validity of the cellular experiments of Trio inhibition. Despite these issues, **52** is advertised as a specific Trio inhibitor.^91^ **49** has even been used in in vivo studies on the effect of inhibiting Rho‐GEF activity of Kalirin 7.^92^

A virtual screen of 4 000 000 compounds from the ZINC library targeting a surface groove of RhoGEF LARG identified Y16 (**53**, Figure 17 A) as a selective inhibitor of LARG over other RhoGEFs, with a *K*_D_ value of 76 nm.^93^ **53** blocked RhoA activity in cells, and inhibited RhoA‐associated breast cancer cell activity synergistically with **48** (Figure 16). However, **53** contains a PAINs motif; the pyrazolidin‐3,5‐diones are potent Michael acceptors.45a, 45b Hence, even though there is evidence for **53** acting on LARG in vitro, off‐target binding should be explored before this compound is considered suitable as a probe or therapeutic.

CPYPP (**54**, Figure 17 A), also containing the pyrazolidin‐3,5‐dione PAINs motif,45a, 45b was identified as an non‐specific inhibitor of DOCK2, a Rac1 GEF, with an IC_50_ value of 23 μm.^94^ STD NMR spectroscopy indicated that **54** bound to the DHR‐2 domain and inhibited DOCK2 GEF activity. Despite low potency, **54** was taken into cells and the effect on DOCK2‐dependent pathways analyzed. However, studies have since found **54** also inhibited the Dbl GEF Trio, indicating it is not specific for the DOCK family of GEFs; it was suggested it could act directly on Rac1, or there could be non‐specificity arising from the reactive PAINs motif.^95^ Despite it being sold as a DOCK sub‐family inhibitor,^96^ the weak potency and presence of a PAIN scaffold call into question the validity of this compound as a useful chemical probe for DOCK2.

Vives et al.^97^ adapted the yeast exchange assay developed by Blangy et al.9a to identify a chemical inhibitor of DOCK5, an important mediator of bone reabsorption in osteoclasts. After screening 2640 heterocyclic compounds, C21 (**55**, Figure 17 B) was identified as an inhibitor of DOCK5. **55** recapitulated Rac1 inhibition phenotypes in cells, and inhibited DOCK5‐mediated nucleotide exchange in a fluorescent exchange assay.^98^ However, only k_obs_ values are listed for a concentration of 50 μm rather than IC_50_ values. **55** also affected DOCK1 and 2 exchange activities, and no exploration of SAR or improvement in potency were made before using **55** extensively in in vitro and in vivo studies. It was later established that **55** acts through a non‐competitive mechanism. DOCK5 dynamically changes to allow Rac1 binding; it is presumed that **55** takes advantage of these changes to bind to DOCK5 and trap in an abortive state in which it can still bind to Rac1 but not promote nucleotide exchange.^95^

### 4.3 RhoGDI/GTPase Complex Inhibitor (Method G)

**Figure 18:**
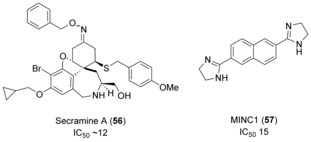
RhoGDI and RhoGAP inhibitors.

Secramine A (**56**, Figure 18) was identified in a screen of 2500 small‐molecule galanthamine mimetics for their ability to inhibit transportation from the endoplasmic reticulum and Golgi apparatus to the plasma membrane of a viral glycoprotein.^99^ **56** also inhibited actin polymerization in vitro, which is controlled by the Cdc42 signaling pathway. The inhibitory effect was attributed to the prevention of Cdc42 recruitment from membranes, thereby blocking activation of Cdc42. This inhibition was dependent on RhoGDI1, which is believed to aid transport of Cdc42 between membranes and the cytosol. Whilst it was hypothesized that **56** stabilizes the RhoGDI1/Cdc42 complex, reducing the amount of Cdc42 available for downstream signaling, evidence that **56** binds to both RhoGDI1 and Cdc42 is still required.

### 4.4 RhoGAP/GTPase Complex Inhibitor (Method H)

MINC1 (**57**, Figure 18) was discovered in a screen of 20 480 compounds to inhibit GAP‐stimulated hydrolysis on Rac1, with an IC_50_ value of 15 μm.^100^ **57** slowed the dissociation of the MgcRacGAP/Rac1 (RhoGAP/GTPase) complex, and it was postulated to stabilize the complex, thereby preventing hydrolysis of GTP. Whilst **57** reduced cell proliferation, consistent with MgcRacGAP inhibition, the authors infer that the different phenotype between **57** inhibition and siRNA‐MgcRacGAP knockout suggests that **57** may affect other cell division factors. However, there are examples of small molecules and siRNA phenotypes not being identical owing to proteins having more than one function.^101^ For example, if a protein acts as a scaffold, then the scaffolding effect could still be seen in small‐molecule inhibition but not in a knockout phenotype, meaning it is not necessarily the case that **57** inhibits other proteins. However, the low potency for the MgcRacGAP Rac1 complex and lack of selectivity characterization of the compound means off‐target effects should be investigated.

## 5 Arf GTPases

### 5.1 Pan‐Arf Inhibitor (Method A)

**Figure 19:**
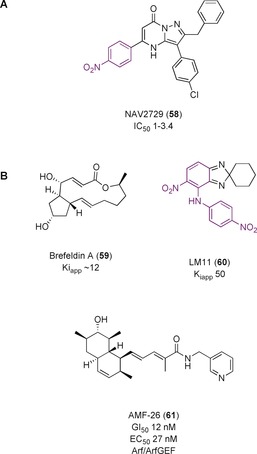
A) NAV2729, a small molecule inhibitor developed for the Arf GTPases. B) Interfacial inhibitors targeting the Arf/ArfGEF subfamily. Toxicophores/structural alerts are shown in purple.

NAV‐2729 (**58**, Figure 19 A) was discovered in a search for a direct inhibitor of Arf6 for the pharmacologic treatment of uveal melanoma.^102^ A HTS of 50 000 compounds resulted in **58** as the most promising candidate for an allosteric, non‐nucleotide competitive probe, with an IC_50_ value of 1–3.4 μm in nucleotide‐exchange assays. It was reported to be selective for Arf6 over other Arf proteins, as well as other small GTPases at concentrations up to 50 μm. Molecular docking studies suggested that **58** bound to Arf6 in the GEF‐binding area, inhibiting GEF interactions. However, Benabti et al. reported that **58** inhibited nucleotide exchange by circa 25 % at concentrations previously reported to cause almost total inhibition in vitro.^103^ IC_50_ values could not be calculated owing to the insolubility of **58** at higher concentrations. It was also more efficient at inhibiting Arf1, despite previously reported as selective for Arf6. Further studies are therefore required before use of **58** as a selective Arf6 probe in cells.

### 5.2 Targeting the ArfGEF/GTPase Complex (Method D)

Several interfacial inhibitors have been developed for the Arf/ArfGEF complexes. Interfacial inhibitors are defined as a drug that targets the interface between two or more different biomolecules that are found in a functional complex.^104^ The drug site is often generated by conformational changes along the interface generated by movements in the macromolecules.

Brefeldin A (BFA, **59**, Figure 19 B) is a fungal macrolide, originally identified as an antibiotic, before the discovery that it inhibited nucleotide exchange on Arf1 made it relevant for targeting GTPase‐related diseases.^105^ It was anticipated that the mechanism of action involved blocking the complex formation by sterically occluding the binding site. Extensive kinetic studies, however, showed that **59** stabilized the Arf1–GDP–Sec7 complex, acting as an uncompetitive inhibitor by trapping the GTPase in an abortive conformation with its GEFs.^106^

**Figure 20:**
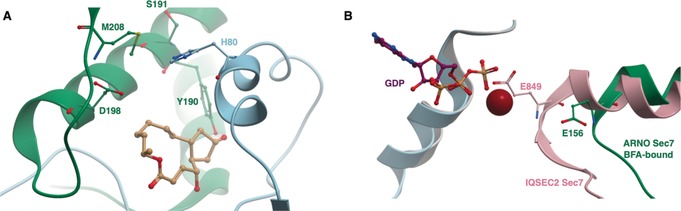
A) PDB 1RE0. Arf1 (blue) bound to BFA‐sensitive ARNO^4M^ (green). The residues critical for BFA binding are labelled. B) Arf1 (blue) bound to BFA‐bound ARNO^4M^ (green) (PDB 1RE0) and Arf1 (grey) bound to IQSEC2 (green) (PDB 6FAE). Binding of BFA prevents reorganization allowing the glutamic finger (E156 in ARNO, E849 in IQSEC2) to be oriented towards GDP, preventing nucleotide exchange.

This hypothesis was confirmed by the crystal structure of **59** bound to the interface of the complex (Figure 20 A).^107^ BFA is active on some, but not all, ArfGEF Sec7 domains; mutational studies identified two pairs of residues (Y190/S191 and D198/M208) in Sec7 and His80 of Arf as essential in conferring sensitivity to **59** (shown in Figure 20 A; ARNO is not BFA‐sensitive without these mutations).106a, 108 The catalytic glutamine finger of the ArfGEF is kept away from the nucleotide in this confirmation, resulting in inhibition of nucleotide exchange (Figure 20 B). A *K*_iapp_ value has been determined at circa 12 μm for Arf1 and ARNO^4M^.^109^ **59** and its derivatives have not progressed to clinical trials owing to poor bioavailability.^110^ **59** established a new method for GTPase inhibition and the selectivity for different members of the Arf and Sec7 families inspired the development of new potent interfacial inhibitors of GTPases and their GEFs.

A pocket near the Arf1/ARNO interface, but remote from the BFA binding site, was targeted using in silico screening by Viaud et al.^111^ in an attempt to inhibit BFA‐insensitive GEFs. LM11 (**60**, Figure 19 B) had a *K*_iapp_ value of circa 50 μm and acted through a non‐competitive mechanism in a nucleotide‐exchange assay. It bound both to Arf1‐GDP and Arf1‐GDP/ARNO, inducing a non‐productive complex. ^1^H–^15^N NMR spectroscopy showed **60** bound near the Arf1 switch 1 region in the targeted flexible pocket. It was sensitive to residue mutations in ARNO, indicating that it interacts with both proteins at their binding interface, and can be considered an interfacial inhibitor. **60** was found in vitro to have specificity for different isoforms of Arf, similar to **59**, providing credence to the interfacial inhibitor hypothesis.

Ohashi et al.^112^ used in silico screening of compounds against a panel of 39 cancer cell lines (JFCR39) for a compound that had a similar pharmacological profile to **59** in an effort to discover an interfacial inhibitor with a chemically distinct scaffold. AMF‐26 (**61**, Figure 19 B) showed Golgi‐body‐disruption activity (EC_50_=27 nm) and cell growth inhibition (GI_50_=12 nm) in BSY‐1 cells. Computational modelling placed **61** in the same interfacial binding site as **59**. In vivo experiments using human breast cancer xenografts showed almost complete regression of the tumor without significant weight loss in the mice upon application of **61**. However, studies by Benabdi et al.^103^ in vitro found that at 15 μm, **61** inhibited a subset of GEFs up to 20–60 %, suggesting a low‐ to mid‐micromolar IC_50_ value. They also showed that **61** was a pan‐ArfGEF inhibitor, and inhibited ARNO, a BFA‐sensitive GEF. This means its inhibitory profile was different to that of **59. 61** has also been found to have anti‐angiogenic properties owing to the inhibition of the VEGF and NF‐κB pathways.^113^ Whilst the promising in vivo efficacy means **61** may be a candidate for further therapeutic development, the low in vitro potency, the undetermined mechanism of action (which could be GEF‐dependent instead of interfacial inhibition) and known lack of selectivity means it is unsuitable for use as a probe.

### 5.3 Inhibitors of ArfGEFs (Method B)

**Figure 21:**
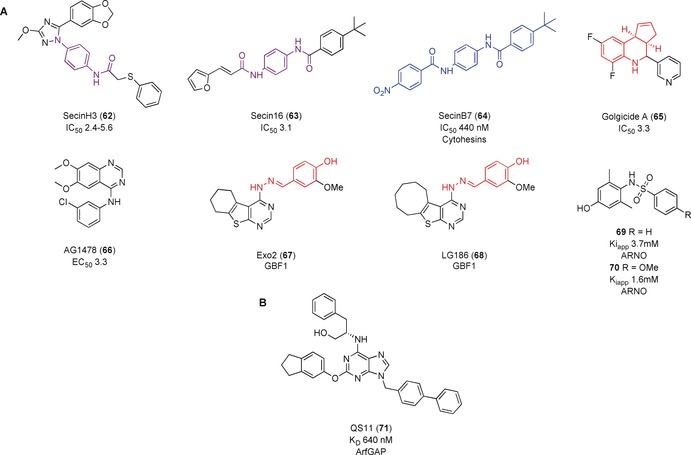
A) ArfGEF inhibitors. B) ArfGAP inhibitor. Structures similar to aggregators shown in blue. PAINs motifs are shown in red. Toxicophores/structural alerts are shown in purple.

The first inhibitor identified against the small ArfGEFs was an RNA aptamer.^114^ A library of 10^15^ RNA sequences was screened against Cytohesin 1 (CYTH1). Selection and evolution of the RNA library resulted in the identification of M69, an aptamer specific for the Sec7 domains of small ArfGEFs. Use of a 5‐fold molar excess of M69 compared to CYTH1 and CYTH2 (ARNO) resulted in 40–50 % inhibition of the exchange activity on Arf1. M69 mimicked a negative mutant of CYTH1 and inhibited cytohesin‐mediated activity in vivo. In further studies, a screening method was established to identify small molecule inhibitors of the cytohesins; small molecules were screened for their ability to displace M69 when bound to a cytohesin protein and adopt its inhibitory activity. SecinH3 (**62**, Figure 21 A) was found in such a screen.^115^ It bound to the Sec7 domains of cytohesins 1–3 (*K*_D_=200–250 nm) and inhibited nucleotide exchange with IC_50_ values between 2.4 and 5.6 μm, although the exemplary ITC graph in the publication showed a very weak signal compared to background. **62** was used in vitro and in vivo to elucidate the role of cytohesins in insulin signaling. However, recent use of **62** by Benabdi et al.^103^ found that it was insoluble at concentrations as low as 15 μm. Benabdi et al. only achieved 30 % inhibition of ARNO against Arf1/6 and so could not calculate an IC_50_ value. **62** was then used as a chemotype template for virtual screening, leading to the discovery of Secin16 (**63**, IC_50_=3.1 μm).^116^ Analogues of **63** screened by FRET resulted in the most potent compound SecinB7 (**64**), which inhibited nucleotide exchange with an IC_50_ value of 440 nm and could inhibit PC9 proliferation (which **62** and **63** could not).^117^ It should be noted, however, that **64** has structural similarity to known aggregators whilst **62** and **63** contain a toxiphoric motif.^22^, 45a

Golgicide A (**65**, Figure 21 A) was identified in a high‐throughput screen as able to protect host cells from the bacterial shiga toxin (IC_50_=3.3 μm). **65** was observed to have similar effects to **59** on the Golgi complex and to be a selective inhibitor for GBF1 over other ArfGEFs.^118^ Molecular modelling predicted **65** to bind and overlap in the same cleft as **59. 65** was predicted to interact with a tripeptide loop present in GBF1, but not BIG1, accounting for the selectivity of **65** for GBF1 seen in cells. Site‐directed mutagenesis of residues in the loop resulted in loss of susceptibility to **65**. This extension has led to the suggestion that GCA acts through an uncompetitive mechanism rather than through interfacial inhibition like **59**. Further information is required to determine the precise mechanism as well as the in vitro potency. It also contains a fused tetrahydroquinoline motif, which has been identified as a PAINs motif that is commonly found in screening campaigns.45a, 45b

Pan et al.^119^ proposed known EGFR inhibitor AG1478 (**66**, Figure 21 A) as an inhibitor of ArfGEF GBF1, following an image‐based assay to identify molecules that induce the Golgi complex to disassemble. The activity of **66** was independent of its EGFR inhibitory activity, and the mechanism of Golgi disassembly was similar to that of interfacial inhibitor **59**. Arf1 pulldown assays showed that **66** blocked Arf1 activity in cells; overexpression and mutation studies suggested that **66** inhibited GBF1 in a mechanism dependent on the Sec7‐domain catalytic activity. A comparison of the activities of **66** and **65** showed **66** was unable to inhibit the replication of enteroviruses, a GBF1‐dependent mechanism, and that overexpression of Arf1 overcame the inhibitory effects of **66** but not **65** or **59**.^120^ This caused the authors to question whether **66** is a genuine direct inhibitor of GBF1. Further validation of the mechanism of action is required before use of **66** as a tool in analyzing the role of GBF1 in cells.

A phenotypic screen identified Exo2 (**67**, Figure 21 A) as a potential inhibitor of GBF1 owing to its ability to block traffic from the endoplasmic reticulum to the Golgi similar to **59** without inducing endosome tabulation.^121^ LG186 (**68**) was developed based on the structure of **67** and the proposed binding site on GBF1.^122^ **68** inhibited Arf activation in cells consistent with GEF inhibition. Weak in vitro inhibition of the Sec7 domain of BIG1, a related GEF, suggested the cellular effects of **68** are due to direct inhibition of GBF1. However, an interfacial inhibition mechanism similar to BFA, or activation of GBF1 could also explain the results, meaning direct measurement of the effect on GBF1 and preferably structural data is required to be sure of the mechanism. They also contain a PAINs motif that should be investigated.

Rouhana et al.^123^ conducted an in silico fragment screening to identify compounds that bind to the catalytic Sec7 GEF domain of ARNO. 33 fragments were predicted to bind within small pockets near predicted hotspots. The potency of the compounds was evaluated using a fluorescent nucleotide‐exchange assay, NMR spectroscopy, and SPR assay. Compounds that aggregated were removed, and PAINs motifs were considered. X‐ray crystal structures were solved for three of the compounds; **69** (Figure 21 A, *K*_iapp_=3.7 mm) bound in the Sec7 domain near key residues required to form interactions in the Arf–ARNO complex, thereby preventing PPIs and complex formation. This compound was chosen for further scaffold optimization and SAR analysis. The most potent follow‐up compounds, **70**, had a *K*_iapp_ value of 1.6 mm and also inhibited Arf1–ARNO complex formation at 1 mm. Despite the low potency of these compounds, the authors demonstrate a strategy for computational fragment screening for PPIs, and the compounds could be useful starting points in the development of potent inhibitors.

### 5.4 ArfGAP Inhibitor (Method I)

QS11 (**71**, Figure 21 B) was identified in a screen of 100 000 heterocycles for its ability to activate Wnt/β‐catenin signaling in cells with an EC_50_ value of 0.5 μm.^124^ ArfGAP1 was identified as the cellular target of **71** using affinity chromatography, with a *K*_D_ value of 640 nm by SPR assay. Levels of Arf1‐GTP and Arf6‐GTP were increased upon **71** application in cells, consistent with ArfGAP inhibition. In MDA‐MB‐231 breast cancer cells, AMAP1, a homologous GAP to ArfGAP1, is essential for migration of the cancer cells. Application of **71** inhibited the migration in a dose‐dependent manner showing it acts a pan‐ArfGAP inhibitor. Further SAR data validated ARFGAP1 as a target of **71** and improved solubility and potency,^125^ although discrepancies between assays lead the authors to note that more comprehensive target identification is required to ensure ArfGAP1 is the major cellular target of **71**.

## 6 Summary and Outlook

In this review we have highlighted the small‐molecule and biologic attempts to modulate GTPase activity by targeting their regulatory proteins GEFs, GAPs, and GDIs. Whilst this strategy is recent, with most of the literature published in the last decade, some molecules with selectivity and potency have been achieved (summarized in Table 4).

**Table 4:** Summary of the small molecules and biologics listed in this review.

	Section	Target	Method	Cmpd ID	Potency	Potency measure	Selectivity targets measured	Protein Cmpd Structure	PAINs?
Small molecules									
**Ras**	4.1	Ras	A	**1**	IC_50_ 0.5 μm	Competition assay	0	No	No
	4.1	Ras	A	**2**	IC_50_ 0.7 μm	Competition assay	0	NMR	No
	4.1	Ras	A	**3**	IC_50_ 90 μm	Fluorescence	0	No	No
	4.1	KRas	A	**4**	IC_50_ 342 μm	Fluorescence	2	X‐Ray	No
	4.1	KRas	A	**5**	*K*_D_ 190 μm	NMR	0	X‐Ray	No
	4.1	Rheb KRas	A	**6**	*K*_D_ 1800 μm *K*_D_ 600 μm	NMR	1	NMR	No
	4.1	KRas	A	**7**	*K*_D_ 5.8 mm	NMR	0	NMR	No
	4.1	Ras	A	**8**	GI_50_ 4‐7 μm	Cell phenotype	0	Docking	No
	4.1	KRas G12C	A	**9**	IC_50_ 1.6 μm	Cellular target engagement	Proteome	X‐Ray	No
	4.1	KRas G12C	A	10**	IC_50_ 120 nm	Cellular target engagement	Proteome	X‐Ray	No
	4.1	RalA,‐B	A	**11**	n.d.	ELISA/cell phenotype	0	Docking	No
	4.1	RalA,‐B	A	**12**	IC_50_ ≈3.5 μm	Cell phenotype	0	NMR	No
	4.1	RalA,‐B	A	**13**	*K*_D_ 4.7 μm *K*_D_ 7.7 μm	SPR ITC	2	NMR	No
	4.3	Sos	B	**14**	*K*_D_ 7 μm	MST	1	Docking	Yes
	4.3	Sos	B	**15**	IC_50_ 5 μm	Fluorescence	1	Docking	No
	4.3	Sos	B	**16**	IC_50_ 32 μm	Fluorescence	1	Docking	No
	4.3	Epac1	B	**17**	IC_50_ 71 μm	Cell phenotype	2	Docking	Yes
	4.3	Epac1	B	**18**	IC_50_ 4 μm	Cell phenotype	2	Docking	Yes
	4.3	Kras/Sos1	B	**19**	n.d^[b]^	SPR	0	Xray	No
	4.3	Sos1	B	**20**	*K*_D_ 450 nm IC_50_ 320 nm	ITC Fluorescence	>100	Xray	No
	4.3	Sos1	B	21*	IC_50_ 21 nm	Fluorescence	5	XRay	No
	4.4	Epac	C	**22**	IC_50_ 8.4 μm	Fluorescence	2	No	No
	4.4	Epac	C	**23**	IC_50_ 4 μm	Fluorescence	2	No	No
	4.4	Epac	C	**24**	IC_50_ 5.9 μm	Fluorescence	2	Docking	No
	4.4	Epac2	C	**25**	IC_50_ 0.4 μm	Fluorescence	2	No	No
	4.4	Epac1 Epac2	C	**26**	IC_50_ 11 μm IC_50_ 2.4 μm	Fluorescence	2	Docking	Yes
	4.4	Epac1	C	**27**	IC_50_ 23 μm	Fluorescence	2	No	No
	4.5	KRas/Sos	D	**28–31**	n.d^[a]^	n/a	0	X‐Ray	No
	4.5	Ras/Sos	D	**32**	EC_50_ 14 μm	Fluorescence	0	X‐Ray	No
	4.5	Ras/Sos	D	**33**	EC_50_ 9 μm	Fluorescence	0	X‐Ray	No
	4.6	p21ras	E	**34**	n.d.^[b]^	Fluorescence	2	No	No
	4.6	KRas	E	**35**	*K*_D_ ≈0.8 μm	SPR	5	No	No
	4.6	Ras	E	**36**	IC_50_ 20 μm	Fluorescence	7	NMR	Yes
	4.6	Ras	E	**37**	IC_50_ 100 μm	Fluorescence	7	NMR	Yes
									
**Rho**	5.1	Rac1	A	**38**	IC_50_ ≈50 μm	Pull‐down assay	6	X‐Ray	No
	5.1	Rac1	A	**39**	IC_50_ 12 μm	G‐LISA^[c]^	2	Docking	Similar to aggregators
	5.1	Rac1	A	**40**	IC_50_ 61 μm	Cell phenotype	1	Docking	No
	5.1	Rac1	A	**41**	IC_50_ 4 μm	Cell phenotype	1	Docking	No
	5.1	Rac1	A	**42**	IC_50_ 2.5 μm	G‐LISA^[c]^	1	Docking	No
	5.1	Rac1/3	A	**43**	IC_50_ 1.1 μm	G‐LISA^[c]^	3	Docking	No
	5.1	Rac1 Cdc42	A	**44**	IC_50_ 103 nm IC_50_ 78 nm	G‐LISA^[c]^	2	Docking	No
	5.1	Rac1 Cdc42	A	**45**	n.d^[b]^	G‐LISA^[c]^	2	No	No
	5.1	Cdc42	A	**46**	n.d^[b]^	G‐LISA^[c]^ Fluorescence	2	No	No
	5.1	Cdc42	A	**47**	*K*_D_ 6.4 μm *K*_D_ 11.4 μm	Fluorescence SPR	2	Docking	No
	5.1	RhoA (RhoB,C)	A	**48**	*K*_D_ 354 nm	MST	5	Docking	No
	5.2	Trio	B	**49**	IC_50_ 116 μm	Fluorescence	2	No	Toxic
	5.2	Trio	B	**50**	IC_50_ 51 μm	Fluorescence	2	No	(May be toxic)
	5.2	Trio	B	**51**	IC_50_ 56 μm	Fluorescence	2	No	(May be toxic)
	5.2	Trio	B	**52**	IC_50_ 76 μm	Fluorescence	3	No	Yes
	5.2	LARG	B	**53**	*K*_D_ 76 nm	MST	5	Docking	Yes
	5.2	DOCK2 DOCK1/5	B	**54**	IC_50_ 23 μm	Fluorescence	5	No	Yes
	5.2	DOCK5 DOCK1/2	B	**55**	n.d.^[b]^	G‐LISA Fluorescence	3	No	No
	5.3	RhoGDI1/Cdc42	G	**56**	IC_50_ ≈12 μm	Cell phenotype	0	No	No
	5.4	MgcRac GAP/ Rac1	H	**57**	IC_50_ 15 μm	Colorimetric	2	No	No
									
**Arf**	6.1	Arf1/ARF6	A	**58**	IC_50_ 1 μm ^[d]^ IC_50_ 3.4 μm ^[d]^	Fluorescence Radiometric	8	Docking	No
	6.2	Arf1 or Arf5/BFA‐ sensitive GEF	D	**59**	*K*_iapp_ ≈12 μm	Fluorescence	>10	XRay	No
	6.2	Arf1‐GDP/ARNO	D	**60**	*K*_iapp_ 50 μm	Fluorescence	7	NMR	No
	6.2	Arf/ ArfGEF	D	**61**	GI_50_ 12 nm EC_50_ 27 nm	Cell phenotype	7	MD	No
	6.3	Cyto‐hesins	B	**62**	IC_50_ 2.4–5.6 μm ^[d]^	Fluorescence	>10	No	No
	6.3	Cyto‐hesins	B	**63**	IC_50_ 3.1 μm	Fluorescence	1	No	No
	6.3	Cyto‐hesins	B	**64**	IC_50_ 440 nm	Fluorescence	0	No	Similarity to aggregators
	6.3	GBF1	B	**65**	IC_50_ 3.3 μm	Cell phenotype	1	No	Yes
	6.3	GBF1	B	**66**	IC_50_ 3.4 μm	Cell phenotype	1	No	No
	6.3	GBF1	B	**67**	n.d	Cell phenotype	2	Docking	Yes
	6.3	GBF1	B	**68**	n.d	Cell phenotype	2	Docking	Yes
	6.3	ARNO	B	**69**	*K*_iapp_ 3.7 mm	Fluorescence	0	X‐Ray	No
	6.3	ARNO	B	**70**	*K*_iapp_ 1.6 mm	Fluorescence	0	X‐Ray	No
	6.4	ARFGAP	I	**71**	*K*_D_ 670 nm	SPR	14	No	No
									
Biologics									
	4.2	Ras	A	**HBS3**	K_D_ 28 μm	Fluorescence polarization	0	NMR	No
	4.2	Ras	A	**SAH‐SOS1**_***A***_	EC_50_ 100–175 nm	Fluorescence polarization	0	NMR	No
	4.2	KRas‐G12D	A	**KRpep‐2 d**	1.6 nm	FRET	2	X‐Ray	No
	4.7	Ras	J	**NF1‐S**	IC_50_ 87 μm	Radiolabeled competition	0	No	No
	6.3	Small ArfGEFs	B	**M69**	K_D_ 16 nm	Filter binding assay	2	No	No

[a] Potency not determined as covalent compounds. [b] Assays conducted but specific potency (IC_50_, *K*_D_, *K*_iapp_) not stated. [c] Cell‐based activation assay for small GTPases. [d] IC_50_ could not be replicated in separate study due to poor solubility. * Suitable for use as a probe in vitro. ** Suitable for use as a probe in vitro and in vivo. Probe criteria: IC_50_ or *K*_D_ <100 nm, >30‐fold selectivity against sequence‐related proteins of the same target family, profiled against relevant off‐targets and on‐target effects in cells at <1 μm concentration.

However, published conclusions are significantly muddied by frequent use of inhibitors without sufficient potency or target validation in cellular studies, which results in misinterpretation of data. In many cases, the authors have found a weak binder and have described the need for optimization and validation of the mechanism of action, but nevertheless the molecules were used by others in biological studies. NSC23766 (**38**), with an in vitro activity of circa 50 μm, is significantly weaker than typical probes (sub‐micromolar). Despite this, it is advertised as a specific inhibitor and has been used to identify Rac1 as a new therapeutic target for the influenza virus, and in gefitinib‐resistant non‐small cell lung cancer.^126^ It has also been reported that NSC23766 acts as a competitive antagonist at muscarinic acetylcholine^127^ and NMDA^128^ receptors in a Rac1‐independent manner, at the same concentrations used for Rac1 inhibition. Moreover, it had critical, Rac1‐independent, off‐target effects in platelets^129^ resulting in the authors concluding the lack of specificity limits their potential without further analysis.

A different problem arises when a small molecule is identified in a cellular assay, and then used in the assumption of it being a specific inhibitor. Issues range from compounds being insufficiently potent, the mechanism of action poorly understood, that off‐target activity had not been sufficiently explored, or that compounds were used without positive or negative controls. An extreme example of this is caffeine, a small molecule well‐documented to bind to other proteins, which was found to inhibit RCC1 RanGEF activity only in the millimolar range, but phenotypes in cellular assays were nevertheless attributed to this inhibition.^130^ The correct and proper use of chemical tools is imperative and misuse could be harmful to the field if the results from poor chemical probes are misinterpreted. It is also recommended that multiple chemotypes are used to examine complex biological phenotypes. Compounds targeting the same protein but with different off‐target effects and chemical scaffolds can be used to investigate whether the resulting modulation is due to inhibition of the desired target. Some of the papers in this review formed conclusions based on a single unvalidated compound. In the worst‐case scenario, it results in a target misidentified in a disease, leading researchers on a wild goose chase, and to the therapeutic strategy of targeting GTPases and their regulators being incorrectly judged to be fruitless.^131^

We have also identified PAINs motifs in many of the compounds targeting the GTPase regulators. Pan assay interference compounds show activity across a range of biochemical, biophysical, and cellular assays and proteins, resulting in false positive identification or interference in in vitro or in vivo assays. It is important to note that by identifying inhibitors containing PAINs, we are not automatically claiming these compounds have been incorrectly identified as inhibitors of the GTPases or their regulators. It is important not to “black box” compounds that contain PAINs and immediately ignore them for future studies; there are FDA improved drugs that contain PAINs motifs45c, 132 and examples of PAINs compounds have been shown crystallographically to bind their putative targets.^133^ Indeed, some of the PAINs compounds listed in this review have persuasive in vitro and in vivo data that correlates with the inhibition of stated targets. However, it is important to be aware of the possibility of the compound being an artefact, so that necessary steps are taken to confirm target selectivity and potency such as robust biophysical measures of binding or co‐crystal structures. Common toxicophores have also been highlighted; whilst these can be acceptable in chemical probes, care should be taken when using compounds for in vivo evaluation or in therapeutic development.^22^ In this review, few publications accommodated for PAINs or toxic moieties in their screens, which when combined with the aforementioned issues in compound design and use is worrying.

The majority of small molecules described in this review are better described as chemical‐probe leads rather than validated chemical probes^131^ and require additional optimization and characterization before they can be used in cellular assays to provide a robust link between phenotype and target. Molecules that meet chemical probe criteria as described by Arrowsmith et al.^134^ are highlighted in Table 4.

Inhibition of protein–protein interactions is notoriously difficult. It was originally conceived that PPIs involved large, flat surfaces with few tractable pockets for small molecule binders. It has now been established that within these surfaces are “hotspots” that theoretically can be targeted with small molecules or biologics. Structural data is often important in identifying these hotspots; the more successful strategies in this review prioritized structural data from the beginning of compound development. In the case of the GTPases, an added difficulty arises owing to the overlap between the binding sites of their regulators and effectors. In this review, it was often seen that for direct GTPase binders, gaining potency through increasing the size of the compound resulted in a loss of selectivity. However, we note that many of the compounds that bind to the GTPase were found in novel pockets, indicating that the surfaces of these proteins may not be as rigid as once believed. Improvements in computational modelling are increasing our understanding of the transient nature of Ras and how this can be targeted with small molecule or biologic therapeutics. As our understanding of how to target PPIs improve, it should become easier to develop inhibitors of interactions between GTPases and their regulators. Indeed, there are now small‐molecule PPI inhibitors in clinical trials and approved drugs when twenty years ago PPIs, much like Ras, were considered undruggable.^135^ The development of PPI‐specific DNA‐encoded libraries is also expected to increase the likelihood of finding a PPI inhibitor, and has already been successful in the development of nanomolar inhibitors against several PPI targets.^136^

However, it is difficult to establish which strategy for targeting the GTPase through their regulators will be the most successful in the future. Most of the compounds identified in this review have low potency, which is not unexpected considering over 60 % are hits discovered directly from initial screens, and only two qualify as probes, making it difficult to determine one method as better than another. The biologics are fairly potent, although they may be hampered by their poor cell permeability. The covalent binders identified are perhaps the most promising as therapeutics, even if they might not be universally applicable to the superfamily owing to lack of accessible cysteine residues. A number of companies are now working on developing covalent inhibitors of the oncoprotein KRas G12C, including the recent announcement of Phase I/II clinical trials for a promising compound.^137^ There is currently little reported preclinical drug discovery on the GTPase regulators. The recent publication^48^ and accompanying patent^138^ from Hillig et al. at Bayer shows that at least one large pharmaceutical company is active in this space.

Despite these obstacles, we believe that the modulation of GTPases, essential proteins in all cellular processes, through their GEF, GAP, and GDI effectors is a very exciting field for drug discovery. Drug discoverers have barely begun to explore the potential of GAPs and GDIs as drug targets, and with care and consideration, it is likely that a therapeutic GTPase/GEF inhibitor is on the horizon.

## Conflict of interest

The authors declare no conflict of interest.

## Biographical Information

*Janine Gray studied Natural Sciences at the University of Cambridge*, *where she received her MSci (specializing in Chemistry) in 2015. Following a year studying Chemistry and Chemical Biology at Harvard University, she joined the groups of Paul Brennan and Frank von Delft for her DPhil at the University of Oxford. Her project involves high‐throughput X‐ray crystallography fragment‐screening for the development of inhibitors for GEF/GTPase complexes, focusing on those implicated in neurodegenerative diseases*.

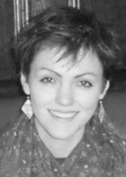

## Biographical Information

*Frank von Delft is jointly Principal Investigator of the Protein Crystallography group in the Structural Genomics Consortium at Oxford University, and Principal Beamline Scientist of beamline I04‐1 at Diamond Light Source synchrotron. After his PhD in protein crystallography with Tom Blundell in Cambridge and postdocs in San Diego at the JCSG and at Syrrx, Inc, he moved to Oxford, where his SGC group has helped solve over 700 crystal structures of human proteins. In late 2012, he partnered with Diamond, setting up the XChem facility that supports dozens of crystal‐based fragment screens annually. His research focus is on methods for moving weak fragment hits to biological potency rapidly and cheaply*.

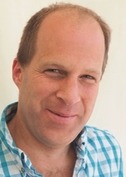

## Biographical Information

*Paul Brennan received his PhD in organic chemistry from the University of California, Berkeley under the mentorship of Paul Bartlett. Following post‐doctoral research with Steve Ley at Cambridge University, and a position at Amgen, Paul accepted a position as medicinal chemistry design lead at Pfizer in Sandwich, UK. In 2011, Paul joined the Structural Genomics Consortium, where he is currently the Professor of Medicinal Chemistry at the SGC and head of chemistry of the Alzheimer's Research UK Oxford Drug Discovery Institute at the University of Oxford. His research is focused on drug discovery for dementia and on finding small‐molecule chemical probes for unexplored protein families*.

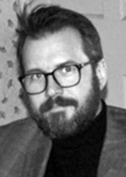

## Supporting information

As a service to our authors and readers, this journal provides supporting information supplied by the authors. Such materials are peer reviewed and may be re‐organized for online delivery, but are not copy‐edited or typeset. Technical support issues arising from supporting information (other than missing files) should be addressed to the authors.

Supplementary
