# Supplementary material for: Targeting the Small GTPase Superfamily through Their Regulatory Proteins
Source: Angew Chem Int Ed Engl. 2020 Jan 30;59(16):6342–66. doi: 10.1002/anie.201900585 (PMC7204875; doi:10.1002/anie.201900585)
Supplement: Supplementary file 1 — Supplementary [file ANIE-59-6342-s001.pdf]

Author Contributions

J.G. Writing - Original Draft: Lead; Writing - Review & Editing: Lead
F.v. Writing - Review & Editing: Supporting
P.B. Writing - Review & Editing: Supporting.
